# Potential Impact of Diet on Treatment Effect from Anti-TNF Drugs in Inflammatory Bowel Disease

**DOI:** 10.3390/nu9030286

**Published:** 2017-03-15

**Authors:** Vibeke Andersen, Axel Kornerup Hansen, Berit Lilienthal Heitmann

**Affiliations:** 1 Focused Research Unit for Molecular Diagnostic and Clinical Research, IRS-Centre Sonderjylland, Hospital of Southern Jutland, Åbenrå 6200, Denmark; 2 Institute of Molecular Medicine, University of Southern Denmark, Odense 5000, Denmark; 3 Department of Veterinary and Animal Sciences, University of Copenhagen, Frederiksberg 1871, Denmark; akh@sund.ku.dk; 4 Research Unit for Dietary Studies, Parker Institute, Frederiksberg 2000, Denmark; berit.lilienthal.heitmann@regionh.dk; 5 Section for General Medicine, Department of Public Health, University of Copenhagen, Copenhagen 1353, Denmark; 6 National Institute of Public Health, University of Southern Denmark, Odense 5000, Denmark

**Keywords:** lifestyle factors, chronic inflammatory diseases, treatment result, treatment response, diet, meat intake, dietary pattern, food, mucosa associated bacteria, epithelium-associated bacteria, microbiome, fibre intake, personalized medicine, mucus, sulphate-reducing bacteria, mucin-degrading bacteria, Western style diet, anti-TNF

## Abstract

We wanted to investigate the current knowledge on the impact of diet on anti-TNF response in inflammatory bowel diseases (IBD), to identify dietary factors that warrant further investigations in relation to anti-TNF treatment response, and, finally, to discuss potential strategies for such investigations. PubMed was searched using specified search terms. One small prospective study on diet and anti-TNF treatment in 56 patients with CD found similar remission rates after 56 weeks among 32 patients with good compliance that received concomitant enteral nutrition and 24 with poor compliance that had no dietary restrictions (78% versus 67%, *p* = 0.51). A meta-analysis of 295 patients found higher odds of achieving clinical remission and remaining in clinical remission among patients on combination therapy with specialised enteral nutrition and Infliximab (IFX) compared with IFX monotherapy (OR 2.73; 95% CI: 1.73–4.31, *p* < 0.01, OR 2.93; 95% CI: 1.66–5.17, *p* < 0.01, respectively). In conclusion, evidence-based knowledge on impact of diet on anti-TNF treatment response for clinical use is scarce. Here we propose a mechanism by which Western style diet high in meat and low in fibre may promote colonic inflammation and potentially impact treatment response to anti-TNF drugs. Further studies using hypothesis-driven and data-driven strategies in prospective observational, animal and interventional studies are warranted.

## 1. Introduction

Inflammatory bowel diseases (IBD), including Crohn’s Disease (CD) and ulcerative colitis (UC), are chronic lifelong illnesses of early onset that substantially affect the life quality of the patients and their families [1,2,3]. Up to 1% of the population in the Western world is affected [4]. The disease burden is continuously rising in the Western world and in newly industrialised countries in Asia, South America, and the Middle East [5]. Thus, more patients will need medical treatment.

The management of IBD has changed significantly during the last two decades [6,7]. The inadequate response to conventional therapy in a large portion of patients and the advances in understanding the role of the inflammatory pathways in IBD [8], has led to the development of bioengineered therapeutic agents targeting key pro-inflammatory molecules. Anti-TNF acts through targeting and neutralising the effect of tumour necrosis factor (TNF-α), thereby diminishing the downstream effects of TNF-α activation. However, the pharmacodynamics of anti-TNF drugs seem to depend on other factors than simply the TNF-binding capacities [8]. Hence, their precise mechanism of action remains unclear. Anti-TNF drugs have proven highly effective for many patients, yet, a significant proportion of the patients do not respond to the treatment (i.e., “primary failures”) or lose the effect over time (i.e., “secondary failures”) [6,7,8]. Despite the introduction of biological treatment of IBD, surgery is still required in 30%–40% of patients with CD and in 25%–30% of patients with UC at some point during their lifetime [9,10]. Sub-therapeutic drug levels and the formation of anti-drug antibodies may decrease their efficacy [11]. Genetics have also been found to define the response to anti-TNF [12,13]. 

Many patients ask their health care professionals about recommendations for dietary intake, and for now we are not able to provide evidence-based answers. Dietary recommendations that can help to improve the outcome of anti-TNF drugs are thus highly warranted by patients. Furthermore, knowledge on diet that supports good treatment response is important for the health care system in order to improve the utilization of the health care resources, as the resources spent for the care of IBD patients are expected to increase exponentially in the coming years [5].

Given the important role of environment on the development of IBD, we hypothesised that lifestyle may impact the effect of anti-TNF drugs. We therefore set out to investigate the current knowledge on the impact of diet on anti-TNF response. Furthermore, we wanted to identify dietary factors that warrant further investigations in relation to anti-TNF treatment response, based on knowledge of their effects on gut inflammation. Finally, we discuss potential strategies for such investigations. For review of diet and risk of IBD please refer to recent papers by Lee et al. and Andersen et al. [14,15]. 

## 2. Materials and Methods

PubMed was searched using the search terms shown in Table 1. In general, the Boolean terms “OR” was used within the groups and “AND” between the groups. The “Similar articles”, “Cited by” function in PubMed and references for key studies were used for finding more articles. Retrieved articles were evaluated for relevance by VA.

## 3. Results

### 3.1. Environmental Factors in IBD

When assessing the effect of lifestyle factors on disease risk, prospective studies are preferred over case-control or cross sectional studies in order to avoid recall bias and bias introduced by lifestyle changes due to the disease itself. Diet seems important [14,15] although strong clinical evidence is scarce [14,15]. In the recent years, prospective studies on diet have emerged. Findings from the European Investigation into Cancer and Nutrition (EPIC) Study and the Nurses’ Health Study (NHS) are particularly noteworthy because of their large well-characterized cohorts [16,17,18,19,20,21,22,23,24,25,26,27,28]. These studies have investigated the involvement of dietary factors such as dietary patterns, vitamin D, dietary fibre, zinc, dairy products, *n*-3 and *n*-6 polyunsaturated fatty acids (PUFA), and protein, particularly animal protein, in IBD development [16,17,18,19,20,21,22,23,24,25,26,27,28]. The increases in IBD seen in developing countries as they adopt a Western lifestyle [5], and the high incidence among immigrants coming from low to high incidence areas, strongly suggest the involvement of other environmental factors such as lifestyle factors in disease aetiology [29]. Non-diet risk factors include family predisposition, birth by caesarean section, smoking, appendectomy, and antibiotics [30]. Smoking is the single most important environmental factor found to be involved in disease development [31]. Smoking increases the risk of CD and aggravates the disease course [31,32], whereas smoking cessation increases the risk of UC, and the risk of flares and the need for drugs in UC [31,32,33]. 

### 3.2. Intestinal Barrier Function and Effects of Anti-TNF

An essential function of the intestinal mucosa is to act as a barrier between luminal contents and the underlying immune system, at the same time allowing absorption of nutrients. The luminal surface of the intestinal mucosa is lined by a mucus layer, a hydrated gel, composed of mucins secreted by goblet cells. In the small intestine, where a large part of nutrient absorption takes place, the mucus layer is discontinuous. In the colon, where most of the gut microbes are present, there is a continuous mucus coat with two layers, an upper “sloppy” layer and a lower adherent layer [34]. In healthy individuals, the mucus prevents large particles and intact microbes from coming into direct contact with the underlying epithelium; thus, the lower adherent layer is generally free from microbes. 

**Figure 1:**
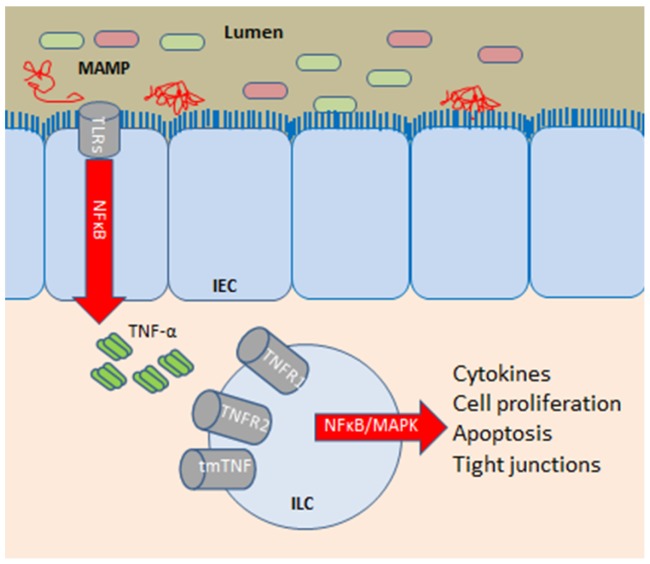
Tumour necrosis factor (TNF-α) is a pro-inflammatory cytokine produced by host cells such as intestinal epithelial cells, macrophages, and lymphocytes, and the principle of anti-TNF-α treatment is the blocking of this [37]. One of the key enteric effector mechanisms of TNF-α production is caused through NF-κΒ, when microbial associated molecular patterns (MAMPs) contained in certain bacteria stimulate the toll-like receptors (TLRs) on a variety of host cells including the enterocytes (intestinal epithelia cells, IEC). The main biological activity of TNF-α is mediated by its binding to TNF receptor type 1 (TNFR1), type 2 (TNFR2), and transmembrane TNF receptors (tmTNFR) [37]. After binding to the receptors, e.g., on innate lymphocytic cells (ILC), TNF-α initiates pro-inflammatory signalling by activation of the MAPKs and NF-κB pathways, leading to the secretion of cytokines (pro-inflammatory TNF-α, IL-1β, IL-6, IFNγ, and anti-inflammatory IL-10), induction of cell proliferation (e.g., via effects on the pluripotent intestinal epithelial stem cells [38]), caspase-8 activation, apoptosis of intestinal cells, and induction of changes in the epithelial expression of tight junction proteins among patients with CD [8]. Anti-TNF treatment neutralizes downstream TNF-α-mediated pro-inflammatory cell signalling and inhibit expression of pro-inflammatory genes. Moreover, TNF inhibitors have been shown to induce apoptosis of TNF-α–producing immune cells, reducing the production of a variety of downstream pro-inflammatory cytokines from these and other cells, and may also induce regulatory macrophages [37,39]. Interestingly, activation of the membrane-bound form of TNF has been suggested to be involved in the downregulation of epithelial apoptosis [40].

In case of defects in the mucus layer, microbes may get into contact with the epithelium. Molecular structures in bacteria known as microbial-associated molecular patterns (MAMPs) stimulate pattern-recognition receptors in the host, most importantly the Toll-like receptors (TLRs), thereby inducing inflammation [35]. Different types of MAMPs stimulate different TLRs dispersed on a variety of different cell types including the enterocytes, and MAMPs are also dispersed and shared between members of the microbiota [36]. Activation of TLRs, including TLR1, TLR2, TLR4, and TLR10 on the intestinal epithelial cells and local innate leukocytes, lead to the activation of the innate immune system via activation of the nuclear factor-κB (NF-κB) signaling pathway [35]. Next, the induction of pro-inflammatory cytokines, such as interleukin (IL)-1, IL-6, tumour necrosis factor-α (TNFα), and interferon-γ (IFNγ), and anti-inflammatory cytokines, such as IL-10, regulates cells of the adaptive immune system (T_H_1, T_H_17, T_H_2, and regulatory T-cells) [35]. Treatment with antibodies targeting TNF neutralizes downstream TNF-α-mediated pro-inflammatory cell signalling and inhibit expression of pro-inflammatory genes (Figure 1).

### 3.3. Impact of Diet on Treatment Results from Anti-TNF Treatment

Exclusive enteral nutrition has been shown to induce remission in some patients with CD, in particular children, whereas, in UC, enteral nutrition had no effect (recently reviewed by Richman [41]). Specialised nutrition-based therapy for direct treatment of CD was first proposed in the 1970s. In patients with mild CD, enteral nutrition monotherapy may be sufficient to induce and maintain clinical remission [42]. A Cochrane meta-analysis on the use of sole enteral nutrition to induce remission in CD, concluded that “the effectiveness of enteral nutrition for the induction of remission in CD is evident from the remission rates” (up to 84%) [41,43]. 

Nutrition therapy has been established in the medical treatment of CD in Japan [44] in addition to conventional drug therapy. Elemental diet therapy (ED) is high-calorie liquid diets containing primarily amino acids that are absorbed by the small intestine without digestion, and was originally developed as food for astronauts who worked for the National Aeronautics and Space Administration in the 1960s [45].

One prospective study on diet and anti-TNF treatment in IBD was identified. The authors compared Infliximab (INF) and ED versus INF and no dietary restrictions in 56 patients with CD that had achieved clinical remission with INF induction therapy [46]. In total, 32 with good compliance were assigned to receive concomitant enteral nutrition, and 24 with poor compliance were assigned to a non-ED group. The 32 patients in the EN group received additional EN therapy with ED (1200–1500 mL) infusion during night-time and a low fat (20–30 g/day) diet during daytime. On an intention-to-treat basis, 25 patients in the EN group (78%) and 16 patients in the non-EN group (67%) remained in clinical remission during the 56-week observation (*p* = 0.51). 

In a retrospective study of primary response in 110 CD patients that received either one single infusion (luminal disease) or three infusions (fistulising disease) with the anti-TNFα drug Infliximab (IFX), 51 patients concomitantly received ED [45]. CD activity index (CDAI) was assessed and CDAI < 150 was defined as clinical remission. The authors reported that 26 out of the 38 patients with inflammatory disease who responded to anti-TNF therapy at week 16, as opposed to 12 out of the 37 of the non-responders, had received concomitant ED treatment (*p* = 0.0026). 

Likewise, Kamata et al. retrospectively studied loss of response in 125 patients with luminal CD treated with scheduled IFX maintenance therapy with a regular dosage [47]. Patients were classified into two groups based on the amount of daily ED intake. The ED group included patients who tolerated 900 kcal/day ED or more, and the non-ED group included those that tolerated less than 900 kcal/day ED at the start of IFX. Furthermore, more patients from the non-ED (32/65) than from the ED (4/24) group were smokers. Twenty-eight patients were categorized as the ED group and 97 patients as the non-ED group. In total, 21 patients developed loss of response in the observational period (mean follow up 799 ± 398 and 771 ± 497 days in the ED and non-ED group). The authors concluded that the ED group was significantly superior to the non-ED group (*p* = 0.049) in sustaining scheduled IFX maintenance therapy.

A recent meta-analysis by Nguyen et al. on the use of IFX monotherapy versus specialised enteral nutrition therapy combination with IFX reviewed 1 prospective study (56 patients) and 3 retrospective studies (in total 295 patients, including the study by Tanaka [45], but not the later study by Kamata [47]) [42]. The daily amount of enteral nutrition ranged from 600 kcal to 1500 kcal/day. Efficacy was measured by clinical response indices. Specialised enteral nutrition therapy with IFX resulted in 109 of 157 (69.4%) patients reaching clinical remission compared with 84 of 185 (45.4%) with IFX monotherapy. In the meta-analysis, there appeared to be more than a two-fold increase in the odds of achieving clinical remission among patients on combination therapy with specialised enteral nutrition and IFX compared with IFX monotherapy (odds ratio (OR) = 2.73; 95% confidence interval (95% CI): 1.73–4.31, *p* < 0.01). Similar results were achieved assessing the numbers that remained in clinical remission after one year (79 of 106 in combination therapy compared to 62 of 126 in monotherapy corresponding to an OR = 2.93; 95% CI: 1.66–5.17, *p* < 0.01). The authors were not able to conclude from the meta-analysis whether or not the type of enteral formula (elemental versus polymeric) made a difference in achieving clinical remission in patients on IFX. The authors stress that the included studies did not fully document the patients’ compliance with the prescribed enteral nutrition formulation, and they concluded that, “Given the limitation of the existing studies, further randomized placebo controlled studies are needed”.

These studies are subject to potential bias due to changes in diet as the consequence of the diseases and their symptoms. These studies need to be replicated in larger prospective, randomised studies before a final conclusion can be reached. 

### 3.4. Impact of Diet on Disease Course and Treatment Results

No other studies on diet and treatment response in IBD patients on anti-TNF are available according to the authors’ knowledge. There is, however, some evidence for the impact of diet on disease course and treatment results from other studies [14,33,41]. 

One study of 191 UC patients, with prospective sampling of diet information using a food frequency questionnaire (FFQ), reported 52 patients had a relapse according to the simple clinical colitis activity index (SCCAI) within one year [48]. A high intake of meat and meat products (particularly red and processed meats), eggs, protein, alcohol, energy, fat, sulphur, and sulphate was associated with an increased likelihood of relapse. Thus, the authors found a strong relationship between a high intake of meat, particularly of red meat and processed meat, and an increased risk of relapse (meat: OR = 3.2; 95% CI: 1.3–7.8, red and processed meat: OR = 5.19; 95% CI: 2.1–12.9). Meat contains essential *n*-6 PUFAs such as linoleic acid. Linoleic acid has been associated with high risk of UC [49]. 

A similar study of 1619 IBD patients from the Crohn’s and Colitis Foundation of America Partners Internet cohort has been performed [50]. Data on diet was sampled by FFQ at entry, and participants were followed for 6 mo. Participants with longer duration of disease, past history of surgery, and past IBD hospitalization ate less fibre. Compared with those in the lowest quartile of fibre consumption, participants with Crohn’s disease in the highest quartile were less likely to have a flare (adjusted OR = 0.58; 95% CI: 0.37–0.90). There was no association between fibre intake and flares in patients with ulcerative colitis (adjusted OR = 1.82; 95% CI: 0.92–3.60) [50].

A randomized controlled trial of 738 patients found no effect of *n*-3 fatty acid supplementation during 1 year in prevention of CD relapse [51], whereas other studies have found *n*-3 fatty acid (fish oil) supplementation to be associated with absence of relapse in CD [52]. In UC, no effect of *n*-3 fatty acid supplementation has been found in a meta-analysis or in a systematic review [41]. The authors of the negative studies have been criticised for use of unsatisfactory omega-3 preparations with variable bioactivity that may have attenuated the observed results of the studies [41,53]. 

A registry study of 3217 patients from a multi-institution cohort with established IBD reported that low plasma Vitamin D status was associated with an increased risk of surgery in CD patients but not in UC patients. Furthermore, CD patients who normalised their vitamin D status had a lower risk of subsequent surgery than those who remained deficient. The authors identified IBD patients with at least one measured plasma 25-hydroxy vitamin D (25(OH)D). Multivariable analysis in CD found that deficient vitamin D status (25(OH)D < 20 ng/mL) was associated with an increased risk of surgery (OR = 1.76; 95% CI: 1.24–2.51) compared with those with normal vitamin D status (25(OH)D > 30 ng/mL). The median lowest plasma 25(OH)D was 26 ng/mL (interquartile range, 17–35 ng/mL). In a subgroup of patients with CD, those who had an initial deficient level of 25(OH)D, but subsequently normalised their values, had a significantly lower likelihood of requiring surgery (OR = 0.56; 95% CI: 0.32–0.98) compared with those who remained deficient. 

These studies, apart from the randomised study, are subject to potential bias, due to changes in diet and serum vitamin D levels as a consequence of the diseases and their symptoms. 

### 3.5. Animal Studies of Potential Relevance for the Impact of Diet on Anti-TNF Treatment Results

Many experimental data suggest the association of dietary factors with gut inflammation in animal models [14,54,55].

#### 3.5.1. A Diet High in Saturated Milk Fat Seems to Promote Colitis

Devkota et al. investigated the effects of three different diets on the enteric microbiota of IL-10-deficient and wild type mice [54]. The diets were low fat diet (LF), polyunsaturated (safflower oil) fat (PUFA) and saturated (milk-derived) fat (MF) diet. MF did not affect the onset and incidence of colitis in wild type mice, but increased onset and incidence in IL-10-deficient mice, driving it from a spontaneous rate of 25%–30% (on LF) to over 60% in a 6-month period. In contrast, the incidence of colitis in IL-10-deficient mice fed PUFA was no different than those fed LF. The colitis seen in mice fed MF was also more severe and extensive. The effects were mediated by MF-promoted taurine-conjugation of hepatic bile acids, which increased the availability of organic sulphur used by sulphate-reducing bacteria like *Bilophila wadsworthia*. When mice were fed a LF diet supplemented with taurocholic acid, but not with glycocholic acid, development of colitis were observed in IL-10-deficient mice. Taurocholic acid in contrast to glycocholic acid can be metabolised to hydrogen sulphide (H_2_S). The data showed that dietary fats, by promoting changes in host bile acid composition, can dramatically alter conditions for gut microbial assemblage, resulting in dysbiosis that can perturb immune homeostasis. 

Interestingly, another mouse study indicated that microbially produced hydrogen sulphide may open the protective mucus barrier and expose the epithelium to potential cytotoxic components, whereas antibiotics (by eliminating sulphate-reducing and mucin-degrading bacteria (e.g., Akkermansia)) blocked microbial sulphide production and thereby maintained the mucus barrier [56]. It was indicated that hydrogen sulphide reduced disulphide bonds in the mucus to trisulphides.

Conflicting results have been found in studies evaluating the effects of *n*-3 and *n*-6 PUFAs in mouse models [57]. However, some studies reported that feeding fish oil to mice decreased production of TNF-α, IL-1β and IL-6 by endotoxin-stimulated macrophages [58].

#### 3.5.2. Vitamin D Deficiency Promotes Diarrhoea

The effect of vitamin D was evaluated in an IL-10-deficient mouse model of IBD [59]. Mice were made vitamin D-deficient, vitamin D-sufficient, or supplemented with active vitamin D (1,25-dihydroxycholecalciferol). Vitamin D–deficient IL-10-deficient mice developed diarrhoea and a wasting disease. In contrast, vitamin D–sufficient IL-10-deficient mice did not develop symptoms. Supplementation with vitamin D (cholecalciferol or 1,25-dihydroxycholecalciferol) significantly ameliorated symptoms, and vitamin D treatment for two weeks blocked the progression and ameliorated symptoms in IL-10-deficient mice with already established IBD. The small intestines from the vitamin D-deficient IL-10-deficient mice were enlarged compared to the vitamin D-sufficient IL-10-deficient mice [59]. Interestingly, administration of 1,25(OH)2D3 was found to suppress the TNF-α pathway [60].

#### 3.5.3. Diet Low in Fibre Confers Susceptibility for Colitis

A recent study, using a simplified model of the human gut microbiome in gnotobiotic mice, found that low dietary intake of fibre increased the susceptibility of colitis by reducing the mucus layer [61]. Mice colonised with the simplified model of the human gut microbiome were fed a fibre-rich or fibre-free diet. Mice fed the fibre-free diet developed increased abundance of host mucosal polypeptide (mucin) degrading bacteria such as *Akkermansia muciniphila* at the cost of bacteria capable of degrading fibre [61]. In addition, gene transcripts from caecal samples showed increased amount of bacterial transcripts encoding enzymes for the metabolism of host polysaccharides such as mucosal O-glucans, at the cost of transcripts encoding for the metabolism of dietary fibre. 

Another study found that oral intake of a specific multi-fibre mix (MFibre), designed to match the fibre content of a healthy diet, counteracted IBD-like intestinal inflammation and weight loss in dextran sodium sulphate (DSS)-treated mice [62]. The authors suggested that the reduction in inflammation might be brought about, at least in part, by the MFibre-induced decrease in inflammatory cytokines, including TNF-α, increase in IL-10, and relative increase in T-regulatory cells in the mesenteric lymph nodes. 

#### 3.5.4. High Salt Diet May Promote Colitis

Mice that received high salt diet (4%) developed a more severe colitis than controls when exposed to trinitrobenzene-sulfonic acid (TNBS) or DSS [63]. Sodium chloride was found to induce T_H_17 cells characterized by up-regulation of TNF-α [64,65].

### 3.6. What is Already Known on Impact of Diet on Gut Inflammatory Mechanisms

Diet is considered to affect gut inflammatory pathways in various ways (Table 2).

For example, dietary fat from meat, such as *n*-6 PUFA, may give rise to arachidonic acid, that may be incorporated in the cell membrane and metabolised to pro- and anti-inflammatory eicosanoids (prostaglandins and leukotrienes) [67]. Dietary protein, in particular from meat, may give rise to the release of marked quantities of branched chain fatty acids and compounds such as ammonia, phenols, and nitric oxide, in addition to sulphide compounds that may have toxic effects on the mucosal epithelium [66]. Dietary fibres from grains, fruit, and vegetables is usually metabolised by the gut microbiome to short chain fatty acids including butyrate, propionate, and acetate. These represent important fuel for the intestinal mucosa and are associated with various anti-inflammatory effects, including e.g. the regulation of G protein-coupled receptors (GPCRs) and mitogen-activated protein kinases (MAPKs) that are of importance for inflammatory processes, and epigenetic regulation of gene transcription by inhibition of histone deacetylases (HDACs) [69]. Vitamin D has impact on the innate and adaptive immune response, as well as the secretion of antimicrobial peptides in the small intestine [68]. Some of these mechanisms may also work in treatment response mechanisms, although the evidence for an involvement in anti-TNF treatment response is scarce. 

### 3.7. New Understanding of Impact of Diet on Gut Inflammation

**Figure 2:**
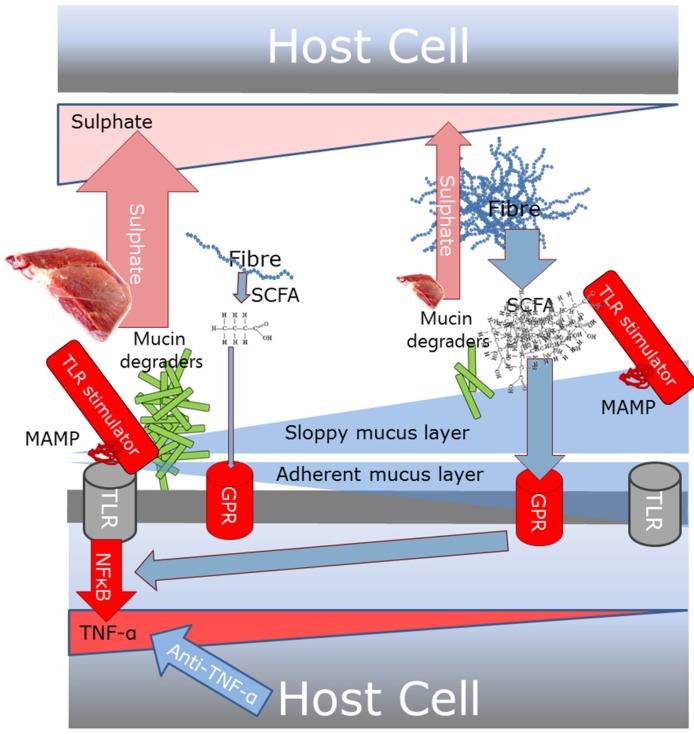
Potential mechanism whereby Western diet high in meat and low in fibre may impact gut inflammation and potentially anti-TNF treatment response. If the enteric mucus layer is thick and intact, the TLR-stimulating MAMP-containing bacteria will be contained in the sloppy mucus layer. If it is thin or disrupted, these bacteria will enter the adherent mucus layer and elicit their TLR stimulation. Dietary sulphate, from e.g. meat, weakens them. Apart from degrading the mucus layer and thereby increasing the potential for MAMP-TLR stimulation, mucus-degrading bacteria release sulphate during the degradation process. A high level of dietary fibres leads to a high level of short chain fatty acids (SCFAs), which stimulates the G protein-coupled receptors (GPRs), thereby counteracting the induction of TNF-α production. Supplementation of a plant fibre diet, as part of the theory, stimulates the mucus layer [41,70].

Low levels of fibre may change the gut microbial metabolism from primarily using microbial derived short chain fatty acids to using mucinous carbohydrates as the main energy source (Figure 2) [61].

Mucolytic bacteria (*Ruminococcus gnavus*, *Ruminococcus torques*, *Akkermansia muciniphila*, and bacteria in the *Bifidobacterium* genus) are defined as mucus-degrading bacteria that are able to grow on mucin as its sole carbon substrate [71]. In IBD, an increased abundance of *Ruminococcus gnavus* and *Ruminococcus torques* and a reduced abundance of *Akkermansia muciniphila* compared to controls have been reported [71]. Degradation of mucus releases free sulphate, which would then become available for utilisation by sulphate-reducing bacteria (like *Bilophila wadsworthia*) for microbially produced hydrogen sulphide [72]. 

High intake of food containing organic sulphur and sulphate additives, such as meat, processed meat, milk, and wine, may increase the amount of sulphate for microbially produced hydrogen sulphide [66,73]. In fact, high amounts of sulphate-reducing bacteria (including *Bilophila wadsworthia*) [72,74] have been found in UC patients.

The resultant hydrogen sulphide from either low intake of fibre, high intake of meat and other sulphur, or both may reduce the disulphide bonds in the mucus network, rendering the mucus layer penetrable to bacteria [72,75]. Then, microbes may reach the epithelium and next activate the TLRs, inducing gut inflammation [35]. 

Additionally, diet may affect the systemic immune response. Intake of low-glycemic index diet was found to lower secretion of TNF-α and IL-6 from stimulated peripheral blood mononuclear cells from obese humans [76].

## 4. Discussion

Diet may impact the treatment response to anti-TNF drugs. We found that evidence-based knowledge on impact of diet on anti-TNF treatment response for clinical use is scarce. One small prospective study on diet and anti-TNF treatment in 56 patients with CD found similar remission rates after 56 weeks among 32 patients with good compliance that received concomitant enteral nutrition and 24 with poor compliance that had no dietary restrictions (78% versus 67%, *p* = 0.51). A meta-analysis of 295 patients found higher odds of achieving clinical remission and remaining in clinical remission among patients on combination therapy with specialised enteral nutrition and Infliximab (IFX) compared with IFX monotherapy (OR 2.73; 95% CI: 1.73–4.31, *p* < 0.01, OR 2.93; 95% CI: 1.66–5.17, *p* < 0.01, respectively). The animal studies provide understanding related to effects of high animal fat and fish oil diets on rates of colitis and cytokine expression. However, caution has to be taken when translating results from therapeutic studies in animals to humans with IBD. Here we hypothesised that Western style diet, high in meat (a rich source of fat, dietary sulphur, and protein) and low in fibre, may seriously impact treatment response in IBD. Such a diet could affect gut microbiome metabolism in a way that promotes gut inflammation and negatively affects medical treatment. There are some previous findings that support such processes in IBD, in particular UC. In fact, high sulphide levels have been found in UC patients [72,75]. Moreover, a defective mucus layer has been associated with UC in humans and colitis in mice [34,77,78]. Additionally, an increased number of epithelium-associated bacteria have been found in IBD, although more consistently in CD than UC [79,80]. Finally, our pharmacogenetics studies of gene-environment interactions in relation to development of colorectal cancer, suggest that intake of red and processed meat interacts with genes encoding TLRs and NF-κB [81], which may offer a potential link to anti-TNF treatment response.

The role of the microbiome in IBD is far from clear. The mucin-degrading bacteria have been suggested to control the abundance of mucosa-associated bacteria by providing substrate for non-mucus degrading bacteria [71]. In addition to their mucin-degrading properties, the mucin-degrading bacteria may have other and diverging abilities. For example, *Akkermansia muciniphila* have been associated with various anti-inflammatory actions [82]. Hence, in obese mice, an increase in number of *Akkermansia muciniphila* has been correlated with increased numbers of goblet cells [83]. Furthermore, *Irgm1* was found to regulate the levels of *Akkermansia muciniphila* in a metabolic mouse model [84]. *IRGM* has been associated with IBD susceptibility and with anti-TNF response in IBD in a pilot study [85] (Hubenthal et al., unpublished). Finally, a mouse study demonstrated that the effect of host genotype on the consumption of mucus glucans was dependent on diet [86]. First, the authors demonstrated impact of a genotype-dependent mucus phenotype (the presence or absence of host fucose) on the gut microbial composition. Next, upon switching to a diet low in polysaccharide, the observed differences seen on a standard diet were lost [86]. The impact of the gut microbiome on the gut immune system thus depends on both the diet and host genetics.

Pharmacogenetic studies may be used for identifying biological mechanisms underlying treatment effects. Our explorative genetic studies of 758 anti-TNF-treated IBD patients suggested that the driving mechanism underlying the inflammation may be individual, and hence, treatment may have to be targeted [12,13]. Our results suggest that IBD patients with genetic variants associated with high TNF inflammatory response were more likely to respond to anti-TNF treatment, whereas IBD patients with genetic variants associated with high IL-1β, IL-6 or INFγ were more likely to be non-responders [13]. Moreover, our ImmunoChip study of 592 anti-TNF-treated IBD patients suggested that *IRGM* and *HNF4G* are involved in defining treatment response. Thus, these results support the involvement of microbes in defining treatment response, as *IRGM* and *HNF4G* encodes proteins involved in autophagy, whereby microbes in the gut are degraded [87,88]. Hence, our exploratory pharmacogenetic studies indicate that the genetic make-up of the host may define the response to anti-TNF treatment and may involve gut-microbe interactions.

Different mechanisms likely define the treatment response in the small intestine and in colon. In the small intestine, a more intimate contact between various dietary components (e.g., gluten) is taking place. In colon, metabolites resulting from microbial degradation of the luminal content may drive the inflammation. Similarly, different mechanisms driving inflammation in CD versus UC may add to the complexity. In accordance, a recent study of genotype-phenotype associations in IBD found that disease location is an intrinsic aspect of disease. The authors demonstrated that IBD is better characterised as three groups (ileal Crohn’s disease, colonic Crohn’s disease, and ulcerative colitis) instead of CD and UC [89].

It has been suggested that plant fibre may be important for restoring the mucus layer [34,79]. Supplementation with complex oligosaccharides such as those present in soluble edible plant fibres was able to inhibit the adherence of pathogen bacteria to epithelial cells in a hen model [70]. Thus, diet modification may represent directions for future treatment and preventative strategies. 

## 5. Conclusions

Diet and other lifestyle factors may potentially impact the treatment response to anti-TNF drugs. However, evidence-based knowledge for clinical use is generally scarce, although vitamin D supplementation to vitamin D-deficient IBD patients seems rational. Our results suggest complex interactions between diet, microbiome, and genetics in defining immune response and treatment response in IBD. Here we propose a mechanism by which a Western style diet high in meat and low in fibre may promote colonic inflammation and potentially affect treatment response to anti-TNF drugs. Further studies of the biological mechanisms of various food items, such as red meat and fibre rich foods, using hypothesis-driven and data-driven explorative strategies in observational studies, animal studies, and interventional studies, may help in understanding how diet may affect treatment results.

## Figures and Tables

**Table 1:** Various combinations of these search terms were used. Both AND (between the groups) and OR (within the groups) were used. The “Similar articles”, “Cited by” functions in PubMed and references for key studies were used for finding more articles.

Group 1	Group 2	Group 3	Group 4	Group 5	Group 6
intestinal bowel disease, Crohn’s disease, ulcerative colitis, inflammatory bowel diseases, chronic inflammatory diseases	lifestyle factor, life style factors, diet, dietary, nutrients, dairy, dietary pattern, food, meat intake, fibre intake, vitamins, high salt diet	tumour necrosis factor, TNF, anti-TNF, treatment response, treatment outcome, treatment result, treatment efficacy, drug, infliximab, IFX, personalised medicine, personalized medicine	sulphate-reducing bacteria, mucolytic bacteria, mucosa-associated bacteria, epithelium-associated bacteria	metabolomics	prospective
IFX, Infliximab					

**Table 2:** Previously suggested mechanisms whereby various diets may affect gut inflammation and potentially anti-TNF treatment response

Food Source	Nutrient	Potential Mechanisms	Reference	
Meat	Protein fermentation	NH_3_ and H_2_S > Mucosal toxicity	Yao, 2016	[66]
	N-6 PUFA	AA pathway > Pro- and anti-inflammatory prostaglandins and leukotrienes		[67]
Fish	Marine n-3 PUFA	EPA and DHA > Altered cell membrane phospholipid fatty acid composition Disruption of lipid rafts Inhibition of pro-inflammatory NFĸB Activation of anti-inflammatory PPARγ Binding to GPR120	Calder, 2015	[58]
Vitamin D	1,25 D vitamin	signalling > Regulation of innate and adaptive immune response Decrease TNF-α secretion in animal models Regulation of antimicrobial peptides	Kamen, 2010	[68]
Vegetables, fruit, cereals, legumes	Fibre	Microbial degradation > SCFA > Fuel for enterocytes Regulation of GPRs and MAPKs Epigenetic regulation of gene transcription by inhibition of HDACs Decrease TNF-α secretion in animal models	Vinolo, 2011	[69]

SCFA; short chain fatty acids, GPRs; G protein-coupled receptors, MAPKs; mitogen-activated protein kinases, HDACs; histone deacetylases, AA; arachidonic acid, PUFA; polyunsaturated fatty acids, EPA; eicosapentaenoic acid, DHA; docosahexaenoic acid, NF-κB; nuclear factor-kappa B, PPARγ; peroxisome proliferator activated receptor γ

## References

[1] Baumgart D.C., Carding S.R. (2007). Inflammatory bowel disease: Cause and immunobiology. Lancet.

[2] Baumgart D.C., Sandborn W.J. (2007). Inflammatory bowel disease: Clinical aspects and established and evolving therapies. Lancet.

[3] Strober W., Fuss I., Mannon P. (2007). The fundamental basis of inflammatory bowel disease. J. Clin. Investig..

[4] Molodecky N.A., Soon I.S., Rabi D.M., Ghali W.A., Ferris M., Chernoff G., Benchimol E.I., Panaccione R., Ghosh S., Barkema H.W. (2012). Increasing incidence and prevalence of the inflammatory bowel diseases with time, based on systematic review. Gastroenterology.

[5] Kaplan G.G. (2015). The global burden of IBD: From 2015 to 2025. Nat. Rev. Gastroenterol. Hepatol..

[6] Dignass A., van Assche G., Lindsay J.O., Lemann M., Soderholm J., Colombel J.F., Danese S., D’Hoore A., Gassull M., Gomollon F. (2010). The second European evidence-based Consensus on the diagnosis and management of Crohn’s disease: Current management. J. Crohns Colitis.

[7] Dignass A., Lindsay J.O., Sturm A., Windsor A., Colombel J.F., Allez M., D’Haens G., D’Hoore A., Mantzaris G., Novacek G. (2012). Second European evidence-based consensus on the diagnosis and management of ulcerative colitis part 2: Current management. J. Crohns Colitis.

[8] Pedersen J., Coskun M., Soendergaard C., Salem M., Nielsen O.H. (2014). Inflammatory pathways of importance for management of inflammatory bowel disease. World J. Gastroenterol..

[9] Bouguen G., Peyrin-Biroulet L. (2011). Surgery for adult Crohn’s disease: What is the actual risk?. Gut.

[10] Hancock L., Mortensen N.J. (2008). How often do IBD patients require resection of their intestine?. Inflamm. Bowel Dis..

[11] Khanna R., Sattin B.D., Afif W., Benchimol E.I., Bernard E.J., Bitton A., Bressler B., Fedorak R.N., Ghosh S., Greenberg G.R. (2013). Review article: A clinician’s guide for therapeutic drug monitoring of infliximab in inflammatory bowel disease. Aliment. Pharmacol. Ther..

[12] Bek S., Nielsen J.V., Bojesen A.B., Franke A., Bank S., Vogel U., Andersen V. (2016). Systematic review: Genetic biomarkers associated with anti-TNF treatment response in inflammatory bowel diseases. Aliment. Pharmacol. Therap..

[13] Bank S., Andersen P.S., Burisch J., Pedersen N., Roug S., Galsgaard J., Turino S.Y., Brodersen J.B., Rashid S., Rasmussen B.K. (2014). Associations between functional polymorphisms in the NF kappa B signaling pathway and response to anti-TNF treatment in Danish patients with inflammatory bowel disease. Pharmacogenom. J..

[14] Lee D., Albenberg L., Compher C., Baldassano R., Piccoli D., Lewis J.D., Wu G.D. (2015). Diet in the pathogenesis and treatment of inflammatory bowel diseases. Gastroenterology.

[15] Andersen V., Olsen A., Carbonnel F., Tjonneland A., Vogel U. (2012). Diet and risk of inflammatory bowel disease. Dig. Liver Dis..

[16] Ananthakrishnan A.N., Khalili H., Higuchi L.M., Bao Y., Korzenik J.R., Giovannucci E.L., Richter J.M., Fuchs C.S., Chan A.T. (2012). Higher predicted vitamin D status is associated with reduced risk of Crohn’s disease. Gastroenterology.

[17] Ananthakrishnan A.N., Khalili H., Konijeti G.G., Higuchi L.M., de Silva P., Fuchs C.S., Willett W.C., Richter J.M., Chan A.T. (2014). Long-term intake of dietary fat and risk of ulcerative colitis and Crohn’s disease. Gut.

[18] Ananthakrishnan A.N., Khalili H., Konijeti G.G., Higuchi L.M., de Silva P., Korzenik J.R., Fuchs C.S., Willett W.C., Richter J.M., Chan A.T. (2013). A prospective study of long-term intake of dietary fiber and risk of Crohn’s disease and ulcerative colitis. Gastroenterology.

[19] Ananthakrishnan A.N., Khalili H., Song M., Higuchi L.M., Richter J.M., Chan A.T. (2015). Zinc intake and risk of Crohn’s disease and ulcerative colitis: A prospective cohort study. Int. J. Epidemiol..

[20] Ananthakrishnan A.N., Khalili H., Song M., Higuchi L.M., Richter J.M., Nimptsch K., Wu K., Chan A.T. (2015). High School Diet and Risk of Crohn’s Disease and Ulcerative Colitis. Inflamm. Bowel Dis..

[21] Chan S.S., Luben R., Olsen A., Tjonneland A., Kaaks R., Lindgren S., Grip O., Bergmann M.M., Boeing H., Hallmans G. (2014). Association between high dietary intake of the *n*-3 polyunsaturated fatty acid docosahexaenoic acid and reduced risk of Crohn’s disease. Aliment. Pharmacol. Therap..

[22] Chan S.S., Luben R., Olsen A., Tjonneland A., Kaaks R., Teucher B. (2013). Body Mass Index and the Risk for Crohn’s Disease and Ulcerative Colitis: Data From a European Prospective Cohort Study (The IBD in EPIC Study). Am. J. Gastroenterol..

[23] Chan S.S., Luben R., van Schaik F., Oldenburg B., Bueno-de-Mesquita H.B., Hallmans G., Karling P., Lindgren S., Grip O., Key T. (2014). Carbohydrate intake in the etiology of Crohn’s disease and ulcerative colitis. Inflamm. Bowel Dis..

[24] John S., Luben R., Shrestha S.S., Welch A., Khaw K.T., Hart A.R. (2010). Dietary *n*-3 polyunsaturated fatty acids and the aetiology of ulcerative colitis: A UK prospective cohort study. Eur. J. Gastroenterol. Hepatol..

[25] De Silva P.S., Luben R., Shrestha S.S., Khaw K.T., Hart A.R. (2014). Dietary arachidonic and oleic acid intake in ulcerative colitis etiology: A prospective cohort study using 7-day food diaries. Eur. J. Gastroenterol. Hepatol..

[26] De Silva P.S., Olsen A., Christensen J., Schmidt E.B., Overvaad K., Tjonneland A., Hart A.R. (2010). An association between dietary arachidonic acid, measured in adipose tissue, and ulcerative colitis. Gastroenterology.

[27] Hart A.R., Luben R., Olsen A., Tjonneland A., Linseisen J., Nagel G., Berglund G., Lindgren S., Grip O., Key T. (2008). Diet in the aetiology of ulcerative colitis: A European prospective cohort study. Digestion.

[28] Opstelten J., Leenders M., Dik V., Chan S., van Schaik F., Siersema P., Bueno-de-Mesquita B., Hart A., Oldenburg B. (2016). Dairy products, dietary calcium and the risk of inflammatory bowel disease: Results from a European prospective cohort investigation. J. Crohns Colitis.

[29] Benchimol E.I., Mack D.R., Guttmann A., Nguyen G.C., To T., Mojaverian N., Quach P., Manuel D.G. (2015). Inflammatory bowel disease in immigrants to Canada and their children: A population-based cohort study. Am. J. Gastroenterol..

[30] Andersen V., Erichsen R., Froslev T., Sorensen H.T., Ehrenstein V. (2013). Differential risk of ulcerative colitis and Crohn’s disease among boys and girls after cesarean delivery. Inflamm. Bowel Dis..

[31] Higuchi L.M., Khalili H., Chan A.T., Richter J.M., Bousvaros A., Fuchs C.S. (2012). A prospective study of cigarette smoking and the risk of inflammatory bowel disease in women. Am. J. Gastroenterol..

[32] Parkes G.C., Whelan K., Lindsay J.O. (2014). Smoking in inflammatory bowel disease: Impact on disease course and insights into the aetiology of its effect. J. Crohns Colitis.

[33] Cosnes J. (2016). Smoking and Diet: Impact on Disease Course?. Dig. Dis..

[34] Merga Y., Campbell B.J., Rhodes J.M. (2014). Mucosal barrier, bacteria and inflammatory bowel disease: Possibilities for therapy. Dig. Dis..

[35] Maloy K.J., Powrie F. (2011). Intestinal homeostasis and its breakdown in inflammatory bowel disease. Nature.

[36] Hansen A.K., Hansen C.H., Krych L., Nielsen D.S. (2014). Impact of the gut microbiota on rodent models of human disease. World J. Gastroenterol..

[37] Nielsen O.H., Ainsworth M.A. (2013). Tumor necrosis factor inhibitors for inflammatory bowel disease. N. Engl. J. Med..

[38] Peterson L.W., Artis D. (2014). Intestinal epithelial cells: Regulators of barrier function and immune homeostasis. Nat. Rev. Immunol..

[39] Cader M.Z., Kaser A. (2013). Recent advances in inflammatory bowel disease: Mucosal immune cells in intestinal inflammation. Gut.

[40] Billmeier U., Dieterich W., Neurath M.F., Atreya R. (2016). Molecular mechanism of action of anti-tumor necrosis factor antibodies in inflammatory bowel diseases. World J. Gastroenterol..

[41] Richman E., Rhodes J.M. (2013). Review article: Evidence-based dietary advice for patients with inflammatory bowel disease. Aliment. Pharmacol. Ther..

[42] Nguyen D.L., Palmer L.B., Nguyen E.T., McClave S.A., Martindale R.G., Bechtold M.L. (2015). Specialized enteral nutrition therapy in Crohn’s disease patients on maintenance infliximab therapy: A meta-analysis. Ther. Adv. Gastroenterol..

[43] Zachos M., Tondeur M., Griffiths A.M. (2007). Enteral nutritional therapy for induction of remission in Crohn’s disease. Cochrane Database Syst. Rev..

[44] Matsueda K., Shoda R., Takazoe M., Hiwatashi N., Bamba T., Kobayashi K., Saito T., Terano A., Yao T. (1995). Therapeutic efficacy of cyclic home elemental enteral alimentation in Crohn’s disease: Japanese cooperative Crohn’s disease study. J. Gastroenterol..

[45] Tanaka T., Takahama K., Kimura T., Mizuno T., Nagasaka M., Iwata K., Nakano H., Muramatsu M., Takazoe M. (2006). Effect of concurrent elemental diet on infliximab treatment for Crohn’s disease. J. Gastroenterol. Hepatol..

[46] Yamamoto T., Nakahigashi M., Umegae S., Matsumoto K. (2010). Prospective clinical trial: Enteral nutrition during maintenance infliximab in Crohn’s disease. J. Gastroenterol..

[47] Kamata N., Oshitani N., Watanabe K., Watanabe K., Hosomi S., Noguchi A., Yukawa T., Yamagami H., Shiba M., Tanigawa T. (2015). Efficacy of concomitant elemental diet therapy in scheduled infliximab therapy in patients with Crohn’s disease to prevent loss of response. Dig. Dis. Sci..

[48] Jowett S.L., Seal C.J., Pearce M.S., Phillips E., Gregory W., Barton J.R., Welfare M.R. (2004). Influence of dietary factors on the clinical course of ulcerative colitis: A prospective cohort study. Gut.

[49] Tjonneland A., Overvad K., Bergmann M.M., Nagel G., Linseisen J., Hallmans G., Palmqvist R., Sjodin H., Hagglund G., Berglund G. (2009). Linoleic acid, a dietary *n*-6 polyunsaturated fatty acid, and the aetiology of ulcerative colitis: A nested case-control study within a European prospective cohort study. Gut.

[50] Brotherton C.S., Martin C.A., Long M.D., Kappelman M.D., Sandler R.S. (2016). Avoidance of Fiber Is Associated With Greater Risk of Crohn’s Disease Flare in a 6-Month Period. Clin. Gastroenterol. Hepatol..

[51] Feagan B.G., Sandborn W.J., Mittmann U., Bar-Meir S., D’Haens G., Bradette M., Cohen A., Dallaire C., Ponich T.P., McDonald J.W. (2008). Omega-3 free fatty acids for the maintenance of remission in Crohn disease: The EPIC Randomized Controlled Trials. J. Am. Med. Assoc..

[52] Turner D., Zlotkin S.H., Shah P.S., Griffiths A.M. (2009). Omega 3 fatty acids (fish oil) for maintenance of remission in Crohn’s disease. Cochrane Database Syst. Rev..

[53] Ferguson L.R., Smith B.G., James B.J. (2010). Combining nutrition, food science and engineering in developing solutions to Inflammatory bowel diseases—Omega-3 polyunsaturated fatty acids as an example. Food Funct..

[54] Devkota S., Wang Y., Musch M.W., Leone V., Fehlner-Peach H., Nadimpalli A., Antonopoulos D.A., Jabri B., Chang E.B. (2012). Dietary-fat-induced taurocholic acid promotes pathobiont expansion and colitis in Il10-/- mice. Nature.

[55] Ejsing-Duun M., Josephsen J., Aasted B., Buschard K., Hansen A.K. (2008). Dietary gluten reduces the number of intestinal regulatory T cells in mice. Scand. J. Immunol..

[56] Ijssennagger N., Belzer C., Hooiveld G.J., Dekker J., van Mil S.W., Muller M., Kleerebezem M., van der Meer R. (2015). Gut microbiota facilitates dietary heme-induced epithelial hyperproliferation by opening the mucus barrier in colon. Proc. Natl. Acad. Sci. USA.

[57] Nanau R.M., Neuman M.G. (2012). Nutritional and probiotic supplementation in colitis models. Dig. Dis. Sci..

[58] Calder P.C. (2015). Marine omega-3 fatty acids and inflammatory processes: Effects, mechanisms and clinical relevance. Biochim. Biophys. Acta.

[59] Cantorna M.T., Munsick C., Bemiss C., Mahon B.D. (2000). 1,25-Dihydroxycholecalciferol prevents and ameliorates symptoms of experimental murine inflammatory bowel disease. J. Nutr..

[60] Zhu Y., Mahon B.D., Froicu M., Cantorna M.T. (2005). Calcium and 1 alpha,25-dihydroxyvitamin D3 target the TNF-alpha pathway to suppress experimental inflammatory bowel disease. Eur. J. Immunol..

[61] Desai M.S., Seekatz A.M., Koropatkin N.M., Kamada N., Hickey C.A., Wolter M., Pudlo N.A., Kitamoto S., Terrapon N., Muller A. (2016). A Dietary Fiber-Deprived Gut Microbiota Degrades the Colonic Mucus Barrier and Enhances Pathogen Susceptibility. Cell.

[62] Hartog A., Belle F.N., Bastiaans J., de Graaff P., Garssen J., Harthoorn L.F., Vos A.P. (2015). A potential role for regulatory T-cells in the amelioration of DSS induced colitis by dietary non-digestible polysaccharides. J. Nutr. Biochem..

[63] Monteleone I., Marafini I., Dinallo V., Di Fusco D., Troncone E., Zorzi F., Laudisi F., Monteleone G. (2017). Sodium chloride-enriched Diet Enhanced Inflammatory Cytokine Production and Exacerbated Experimental Colitis in Mice. J. Crohns Colitis.

[64] Kleinewietfeld M., Manzel A., Titze J., Kvakan H., Yosef N., Linker R.A., Muller D.N., Hafler D.A. (2013). Sodium chloride drives autoimmune disease by the induction of pathogenic TH17 cells. Nature.

[65] Wu C., Yosef N., Thalhamer T., Zhu C., Xiao S., Kishi Y., Regev A., Kuchroo V.K. (2013). Induction of pathogenic TH17 cells by inducible salt-sensing kinase SGK1. Nature.

[66] Yao C.K., Muir J.G., Gibson P.R. (2016). Review article: Insights into colonic protein fermentation, its modulation and potential health implications. Aliment. Pharmacol. Ther..

[67] Wang D., Dubois R.N. (2010). Eicosanoids and cancer. Nat. Rev. Cancer.

[68] Kamen D.L., Tangpricha V. (2010). Vitamin D and molecular actions on the immune system: Modulation of innate and autoimmunity. J. Mol. Med. (Berl. Ger.).

[69] Vinolo M.A., Rodrigues H.G., Nachbar R.T., Curi R. (2011). Regulation of inflammation by short chain fatty acids. Nutrients.

[70] Parsons B.N., Wigley P., Simpson H.L., Williams J.M., Humphrey S., Salisbury A.M., Watson A.J., Fry S.C., O’Brien D., Roberts C.L. (2014). Dietary supplementation with soluble plantain non-starch polysaccharides inhibits intestinal invasion of Salmonella Typhimurium in the chicken. PLoS ONE.

[71] Png C.W., Linden S.K., Gilshenan K.S., Zoetendal E.G., McSweeney C.S., Sly L.I., McGuckin M.A., Florin T.H. (2010). Mucolytic bacteria with increased prevalence in IBD mucosa augment in vitro utilization of mucin by other bacteria. Am. J. Gastroenterol..

[72] Gibson G.R., Macfarlane G.T., Cummings J.H. (1993). Sulphate reducing bacteria and hydrogen metabolism in the human large intestine. Gut.

[73] Windey K., de Preter V., Louat T., Schuit F., Herman J., Vansant G., Verbeke K. (2012). Modulation of protein fermentation does not affect fecal water toxicity: A randomized cross-over study in healthy subjects. PLoS ONE.

[74] Jia W., Whitehead R.N., Griffiths L., Dawson C., Bai H., Waring R.H., Ramsden D.B., Hunter J.O., Cauchi M., Bessant C. (2012). Diversity and distribution of sulphate-reducing bacteria in human faeces from healthy subjects and patients with inflammatory bowel disease. FEMS Immunol. Med. Microbiol..

[75] Ijssennagger N., van der Meer R., van Mil S.W. (2016). Sulfide as a Mucus Barrier-Breaker in Inflammatory Bowel Disease?. Trends Mol. Med..

[76] Kelly K.R., Haus J.M., Solomon T.P., Patrick-Melin A.J., Cook M., Rocco M., Barkoukis H., Kirwan J.P. (2011). A low-glycemic index diet and exercise intervention reduces TNF(alpha) in isolated mononuclear cells of older, obese adults. J. Nutr..

[77] Sheng Y.H., Hasnain S.Z., Florin T.H., McGuckin M.A. (2012). Mucins in inflammatory bowel diseases and colorectal cancer. J. Gastroenterol. Hepatol..

[78] Chen S.J., Liu X.W., Liu J.P., Yang X.Y., Lu F.G. (2014). Ulcerative colitis as a polymicrobial infection characterized by sustained broken mucus barrier. World J. Gastroenterol. WJG.

[79] Flanagan P., Campbell B.J., Rhodes J.M. (2011). Bacteria in the pathogenesis of inflammatory bowel disease. Biochem. Soc. Trans..

[80] Strober W. (2013). Impact of the gut microbiome on mucosal inflammation. Trends Immunol..

[81] Kopp T.I., Andersen V., Tjonneland A., Vogel U. (2015). Polymorphisms in NFκB1 and TLR4 and interaction with dietary and life style factors in relation to colorectal cancer in a Danish prospective case-cohort study. PLoS ONE.

[82] Shin N.R., Lee J.C., Lee H.Y., Kim M.S., Whon T.W., Lee M.S., Bae J.W. (2014). An increase in the Akkermansia spp. population induced by metformin treatment improves glucose homeostasis in diet-induced obese mice. Gut.

[83] Li J., Lin S., Vanhoutte P.M., Woo C.W., Xu A. (2016). Akkermansia Muciniphila Protects Against Atherosclerosis by Preventing Metabolic Endotoxemia-Induced Inflammation in Apoe-/- Mice. Circulation.

[84] Greer R.L., Dong X., Moraes A.C., Zielke R.A., Fernandes G.R., Peremyslova E., Vasquez-Perez S., Schoenborn A.A., Gomes E.P., Pereira A.C. (2016). Akkermansia muciniphila mediates negative effects of IFN gamma on glucose metabolism. Nat. Commun..

[85] Jostins L., Ripke S., Weersma R.K., Duerr R.H., McGovern D.P., Hui K.Y., Lee J.C., Schumm L.P., Sharma Y., Anderson C.A. (2012). Host-microbe interactions have shaped the genetic architecture of inflammatory bowel disease. Nature.

[86] Kashyap P.C., Marcobal A., Ursell L.K., Smits S.A., Sonnenburg E.D., Costello E.K., Higginbottom S.K., Domino S.E., Holmes S.P., Relman D.A. (2013). Genetically dictated change in host mucus carbohydrate landscape exerts a diet-dependent effect on the gut microbiota. Proc. Natl. Acad. Sci. USA.

[87] Delgado M., Singh S., De Haro S., Master S., Ponpuak M., Dinkins C., Ornatowski W., Vergne I., Deretic V. (2009). Autophagy and pattern recognition receptors in innate immunity. Immunol. Rev..

[88] Randall-Demllo S., Chieppa M., Eri R. (2013). Intestinal epithelium and autophagy: Partners in gut homeostasis. Front. Immunol..

[89] Cleynen I., Boucher G., Jostins L., Schumm L.P., Zeissig S., Ahmad T., Andersen V., Andrews J.M., Annese V., Brand S. (2016). Inherited determinants of Crohn’s disease and ulcerative colitis phenotypes: A genetic association study. Lancet.

